# Effect of Iodothyronines on Thermogenesis: Focus on Brown Adipose Tissue

**DOI:** 10.3389/fendo.2018.00254

**Published:** 2018-05-23

**Authors:** Federica Cioffi, Alessandra Gentile, Elena Silvestri, Fernando Goglia, Assunta Lombardi

**Affiliations:** ^1^Department of Sciences and Technologies, University of Sannio, Benevento, Italy; ^2^Department of Biology, University of Naples Federico II, Naples, Italy

**Keywords:** thyroid hormone, metabolism, 3,5-diiodo-l-thyronine, thermogenesis, brown adipose tissue

## Abstract

Thyroid hormones significantly influence energy expenditure by affecting the activity of metabolic active tissues, among which, mammalian brown adipose tissue (BAT) plays a significant role. For a long time, the modulation of BAT activity by 3,3′,5-triiodo-l-thyronine (T3) has been ascribed to its direct actions on this tissue; however, recent evidence indicates that T3, by stimulating specific brain centers, activates the metabolism of BAT *via* the sympathetic nervous system. These distinct mechanisms of action are not mutually exclusive. New evidence indicates that 3,5-diiodo-l-thyronine (3,5-T2), a thyroid hormone derivative, exerts thermogenic effects, by influencing mitochondrial activity in metabolically active tissues, such as liver, skeletal muscle, and BAT. At the moment, due to the absence of experiments finalized to render a clear cut discrimination between peripheral and central effects induced by 3,5-T2, it is not possible to exclude that some of the metabolic effects exerted by 3,5-T2 may be mediated centrally. Despite this, some evidence suggests that 3,5-T2 plays a role in adrenergic stimulation of thermogenesis in BAT. This mini-review provides an overview of the effects induced by T3 and 3,5-T2 on BAT thermogenesis, with a focus on data suggesting the involvement of central adrenergic stimulation. These aspects may reveal new perspectives in thyroid physiology and in the control of energy metabolism.

## Introduction

In mammals and in homeotherms, a tight control of heat production allows maintenance of a constant core temperature despite variations in environmental temperature. Heat production is customarily divided into obligatory and facultative/adaptative thermogenesis. Obligatory thermogenesis represents constitutive heat production, normally resulting from sustaining vital functions; it is sufficient to maintain body temperature of animals at thermoneutrality. When ambient temperature descends below thermoneutral temperature (that differs between mammal species), heat-saving and heat-producing mechanisms are activated. Indeed, heat-saving mechanisms (pilo-erection, vasoconstriction, adoption of a curled posture, immobility) are limited, and an additional heat is promptly produced by a large energy consuming process, such as shivering, and then substituted by a long-lasting activation of more efficient heat generating metabolic mechanisms, occurring principally in brown adipose tissue (BAT) (1).

Recently, emerging novel aspects have renewed the interest for BAT as a potential target for the treatment of human obesity and related diseases (2, 3) since functional BAT has been detected in adult human (4–6), where its amount correlates positively with resting metabolic rate and inversely with body mass index (7). BAT can utilize blood glucose and lipid, thus improving glucose metabolism and lipid profiles in some conditions (8, 9). Moreover, when specifically stimulated, BAT-precursor cells placed in white adipose tissue (WAT) can differentiate to beige/brite cells instead of white adipocytes (10, 11). Thus, studies on the mechanism underlying BAT activation as well as its hormonal regulation are still ongoing.

Thyroid hormones (TH) play a crucial role in stimulating both obligatory and adaptive thermogenesis (12, 13), with BAT thermogenesis being a key contributor of the latter.

Although thyroid thermogenesis is known for more than a century (14), new innovative concepts are emerging; these regard the involvement of the hypothalamus in TH induced-thermogenesis, the ability of TH to induce the browning of WAT, and the identification of 3,5-diiodo-l-thyronine (3,5-T2) as an active TH derivative able to enhance BAT thermogenesis.

Here, we provide an overview on the effects induced by TH and 3,5-T2 on BAT thermogenesis, pointing the attention on aspects suggesting the involvement of central adrenergic stimulation.

## BAT-Mediated Thermogenesis

**Figure 1:**
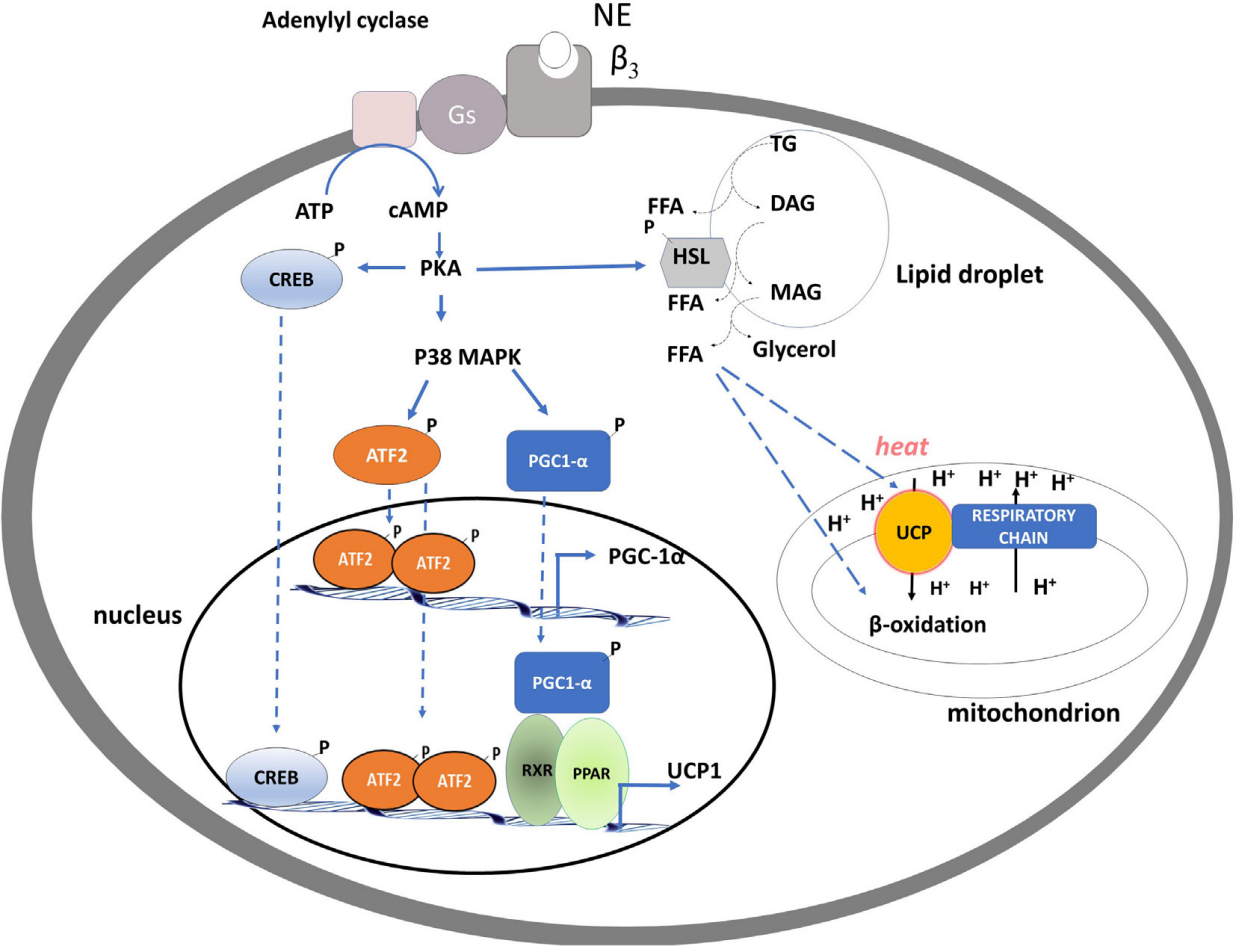
Schematic representation of processes activated by noradrenaline in brown adipocytes leading to brown adipose tissue thermogenesis, mitochondrial biogenesis, and fatty acid oxidation. PKA activation of CREB and p38 MAP kinase leads to an enhancement of the transcription of uncoupling protein-1 (UCP1) and PGC-1α genes. PKA, by phosphorylating hormone sensible lipase leads to the hydrolysis of triglycerides. Free fatty acids released are used as fuel substrate at the mitochondrial levels and as UCP1 activator.

The single heat-producing unit of BAT is the brown adipocyte, it contains triglycerides within multiple small vacuoles and numerous mitochondria. Each cell interacts with noradrenergic fibers of sympathetic nervous system (SNS) and is surrounded by capillaries. When an increased rate of heat production is needed, a signal is transmitted *via* the SNS to each brown adipocyte. The released norepinephrine (NE) primarily binds to the brown adipocytes’ β3 adrenergic receptors and activates intracellular signaling that leads to the hydrolysis of triglycerides and the release of free fatty acids (FFAs) (1). At the mitochondrial level, FFAs are then oxidized, thus furnishing reduced substrates for the respiratory chain that actively pumps protons from the matrix to the inner membrane space and generates proton motive force. In BAT, uncoupling protein-1 (UCP1) is directly activated by FFAs and mediates the re-entry of protons into the matrix, not associated to ATP synthesis, leading to (i) energy dissipation contained in the proton motive force as heat and (ii) substrate oxidation, uncoupled by the synthesis of ATP. Thus, in BAT, UCP1 “converts fat to heat” (Figure 1).

Prolonged exposure to cold and β-adrenergic stimuli triggers a trophic response through activation of mitochondriogenesis, contributes to the increase of the thermogenic capacity of brown adipocytes, and promotes the browning processes of WAT (15).

The molecular events involved in the NE-induced BAT thermogenesis include activation of the cAMP/PKA signaling pathway, downstream stimulation of the p38α MAPK, and recruitment and p38-dependent activation of PGC-1α [peroxisome proliferator-activated receptor γ coactivator-1] (16) (Figure 1).

In BAT, PGC-1α coordinates the expression of genes that stimulate mitochondrial biogenesis and a thermogenic program (17). Indeed, mice lacking PGC-1α are extremely cold sensitive (18, 19), because of a defective thermogenesis, likely due to an impaired mitochondrial program for fatty-acid β-oxidation and electron transport, accompanied by reduced induction of UCP1 and type 2-deiodinase (D2) (see below). In nuclei, PGC-1α coactivates nuclear respiratory factors-1 and -2, which regulate expression of a nuclear-encoded transcription factor essential for replication, maintenance, and transcription of mitochondrial DNA: mitochondrial transcription factor A (mt-TFA). Intra-mitochondrial PGC-1α associates with nucleoids and forms a multiprotein complex with mt-TFA at the mitochondrial DNA transcription start site, thus having a putative role as a transcriptional coactivator of mtTFA (20).

## TH and BAT Thermogenesis

Thyroid hormones are essential for the full thermogenic response of BAT, and normal systemic thyroid status is essential for cold-induced adaptive thermogenesis. The thermogenic response of BAT to TH is the result of the synergistic interactions of the hormones with the SNS (21, 22).

Brown adipocytes express both TH receptors alpha (TRα) and beta (TRβ) that control distinct and fundamental pathways for adequate BAT thermogenesis. TRα mediates synergism between TH signaling and the SNS, whereas TRβ is involved in T3 mediated regulation of UCP1 transcription (23, 24). Indeed, the disruption of TRβ-mediated signaling leads to defective adaptive thermogenesis and reduced UCP1 expression (24, 25), while the TRβ agonist GC-1, when applied in association with NE to isolated brown adipocytes, increases UCP1 expression but NE responses result blunted (24).

This TRβ-dependent mechanism is also crucial to induce UCP1 expression in WAT, thus suggesting a role for TH-signaling in the “browning” phenotype of WAT (26).

The intracellular action of TH is regulated by the amount of cellular T3 available for receptor binding, with deiodinase 2 (D2) playing a crucial role. Brown adipocytes express D2, a TH activating enzyme that catalyzes the deiodination of T4 to T3. Within a few hours of cold exposure, because of D2 activation, intracellular T3 levels increase threefold, resulting in higher T3 receptor occupancy nearly reaching saturation (27–29).

D2 is also crucial for the synergism between TH and NE signaling (22), as supported by the evidence that transgenic D2 null mice show cold intolerance, despite normal plasma T3 concentrations (30). NE leads to an enhancement of D2 levels by promoting its de-ubiquitination and by enhancing its gene transcription (31). As a result, tissue levels of T3 increase, thus amplifying the SNS-induced effects, such as lipolysis and stimulation of the UCP1 gene transcription.

Hyperthyroidism stimulates both basal and facultative thermogenesis. Specifically, alongside enhanced thermogenesis hyperthyroid mice displayed increased BAT mass (32, 33), mitochondrial content, oxidative capacity, and UCP1 protein levels (33). T3 also increases nuclear and mitochondrial PGC-1α levels, pointing to a coordinative effect of this iodothyronine in these two organelles, thus activating mitochondrial biogenesis and BAT thermogenesis. Hyperthyroidism also induces “browning” of WAT (32). In addition to TH, thyrotropin receptor signaling is also involved in BAT formation in the hyperthyroid state, probably *via* upregulation of browning factors such as PRDM6, PGC-1α, and UCP1 (34).

In hyperthyroid rodents, compensatory mechanisms are triggered to limit the response of BAT to both NE and TH. Specifically, an excess of thyroxine (i) promotes ubiquitination and degradation of D2, thus protecting BAT from the elevated serum levels of TH and (ii) reduces β3 receptor density, thus toning down sympathetic stimulation (35, 36). Conversely, hypothyroidism reduces obligatory thermogenesis, accompanied by a compensatory increase in BAT stimulation (21). In fact, hypothyroid BAT shows signs of adrenergic stimulation, such as enhancements of NE levels (32) and sympathetic innervations (33). Despite of this, the lack of T3 limits the thermogenic response of the tissue to the NE stimulation (37). Indeed, cAMP generation is greatly reduced in isolated brown adipocytes obtained from hypothyroid animals as a result of modifications of the receptor, its interaction with Gi proteins, and resulting adenylyl cyclase levels (35, 36, 38). In line with this, hypothyroid animals present impaired BAT thermogenesis, therefore, developing severe hypothermia resulting in death after a few days of exposure to cold (39). Lower BAT activity/thermogenesis despite enhanced sympathetic tones in hypothyroidism has been recently confirmed by a technique termed “*in vivo* small animal 18F-FDG PET/MR” (32). Moreover, histological analysis revealed that BAT from hypothyroid animals shows more lipid-depleted adipocytes, typified by an increased number of unilocular adipocytes, similar to what is observed in white adipocytes (32, 33).

Interestingly, in hypothyroid mice, a compensatory “browning” of WAT seems to occur as a response to the decreased heat production due to BAT inactivity. Markers for this event are increased expression levels of brown fat specific genes such as UCP1 and Cidea, and the multilocular UCP1-positive phenotype of some adipocytes in both iWAT and gWAT (32, 40).

## Central Effect of TH in Activation of BAT

Recent studies revealed that T3 is a central inducer of BAT by directly stimulating the hypothalamic pathway (41). In definite hypothalamic centers, and specifically in Sf1 neurons of ventromedial nucleus (VHM) (42, 43), T3 selectively increases *de novo* lipogenesis, leading to the activation of the SNS and the induction of BAT (41). T3-induced lipogenesis is mediated by AMP-activated protein kinase (AMPK), a key kinase regulating lipid metabolism that, when activated by phosphorylation, switches-on fatty acid oxidation rate and switches-off lipogenesis (44).

Following intracerebroventricular administration, T3 causes a rapid dephosphorylation of AMPK. The crucial role played by AMPK in the T3-induced BAT thermogenesis through the SNS is shown in experiments of VMH-selective genetic ablations of AMPK or TH receptors: AMPK ablation increases the sympathetic BAT tone and subsequent BAT activation, which is blunted by inhibition of β3 adrenergic receptor activity in BAT (41). Ablation of TH receptors in hyperthyroid rats significantly inhibits BAT thermogenesis (41–43).

**Figure 2:**
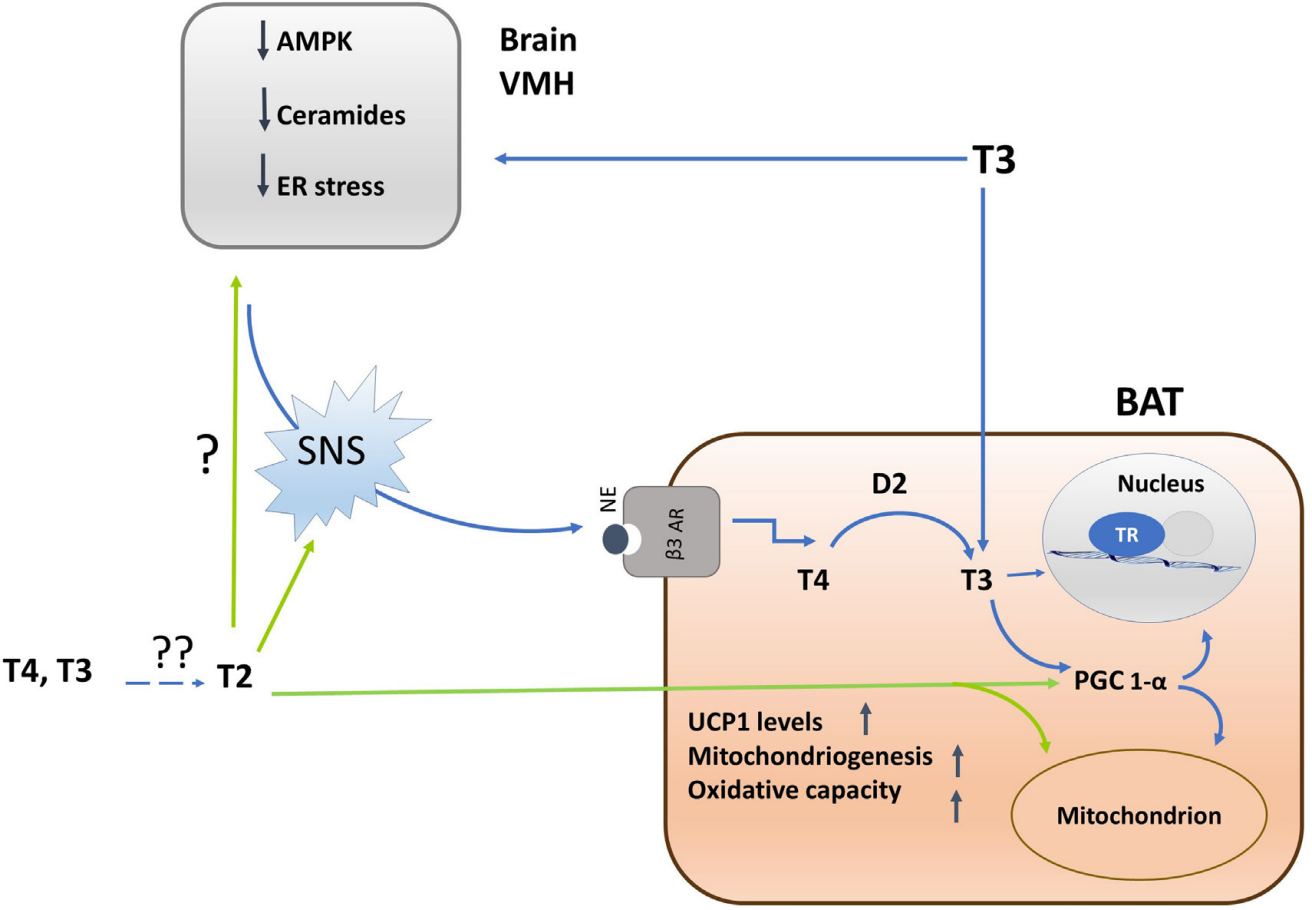
Schematic representation of the mechanisms by which iodothyronines (T3 and 3,5-T2) activates brown adipose tissue (BAT) thermogenesis and of the interrelations between central and direct effects of iodothyronines on the tissue. Single question point indicates that, despite of the fact that 3,5-T2 enhances BAT adrenergic tone, now, there are no experiments indicating whether this effect is brain-mediated or not. Double question points indicate that there are unresolved questions such as: where is 3,5-T2 coming from? Is it coming from the circulation or is it locally formed by deiodination? Is D2 involved? Experimental evidences are needed.

The mechanism linking T3-induced AMPK de-phosphorylation in VHM to the activation of SNS seems related to the ability of AMPK to influence ceramide levels as well as to endoplasmatic reticulum (ER) stress (42, 43). Indeed, at the hypothalamic level, ceramide-induced lipotoxicity triggers ER stress and leads to a decreased sympathetic tone in BAT, thus impairing BAT thermogenesis (45). Thus, T3-induced hypothalamic AMPK dephosphorylation leads to a decrease in ceramide synthase activity, and consequently ceramides levels, resulting in a reduction of ER stress (see Figure 2).

In BAT, following ICV administration of T3, the expression of thermogenic markers (UCP1, PGC1α, D2, hormone sensitive lipase, lipoprotein lipase) occurs in association with a decreased lipid droplet (LD) content as well as an enhancement of the mitochondrial size (41–43). Furthermore, an increase in the BAT-mediated uptake of fatty acids and their subsequent utilization as fuel substrates at the mitochondrial level is observed, plausibly mediated by increased activity of the AMPK signaling pathways (42, 43).

Interestingly, the effects of ICV administration of T3 on energy expenditure, thermogenesis, and body weight are abolished in UCP1-deficient mice (46), suggesting the importance of BAT in TH metabolic regulation.

Another significant effect to consider is the ability of TH to regulate WAT browning through a central mechanism, since ICV infusion of T3 increases the browning of iWAT (46).

## 3,5-T2: A Thyroid Hormone Derivative That Enhances BAT Thermogenesis

The research field concerning the control of energy metabolism by the thyroid is no longer restricted to T3 since growing evidence indicates that some of its derivatives could have biological effects. Among these, 3,5-T2 plays a significant role [for review, see Ref. (47–49)].

3,5-T2 influences the activity of metabolically active tissues such as skeletal muscle, liver, and BAT. Some of the effects induced by this iodothyronine are manifested in a short-term within 1 h of its administration, and experimental evidence indicates that mitochondria are a direct target of 3,5-T2 (47, 48, 50). The ability of 3,5-T2 to exert a calorigenic effect when injected into rats (33, 39, 51–54) suggests that 3,5-T2 could be involved in the regulation of energy metabolism in physiological situations requiring a surplus of energy expenditure, such as cold exposure. Such a possibility is substantiated by the evidence that hypothyroid rats do not survive in the cold (4°C), but their survival is improved by 3,5-T2 administration. Indeed, enhanced oxidative capacity of metabolically active tissues, among these BAT, underlies the ability of 3,5-T2 to improve cold tolerance of hypothyroid rats (55).

When administered intraperitoneally to hypothyroid rats housed at thermoneutrality, 3,5-T2 reverses the “white-like” appearance of brown adipocytes characteristic of such animals (see above), enhancing the percentage of multilocular versus unilocular cells. 3,5-T2 also decreases the diameter of LDs and increases the tissue’s mitochondrial content, diagnostic for BAT activation (33).

Chronic intraperitoneal 3,5-T2 administration to hypothyroid rats improves maximal tissue oxidative capacity by increasing the activity of cytochrome c oxidase (COX) (33, 39, 51). Within the mitochondrial respiratory chain, apart from transferring electrons to oxygen molecules, COX actively pumps protons from the mitochondrial matrix to the intermembrane space, thus contributing to generation of the proton-motive force, which in BAT, due to the presence of UCP1, is dissipated as heat at the expense of ATP synthesis. The ability of 3,5-T2 to enhance COX activity in BAT of hypothyroid rats seems to be the result of a dual mechanism: (1) an increase in tissue content of mitochondria, (2) a direct effect of the iodothyronine on the enzyme (33). In fact, the stimulatory effect of 3,5-T2 occurs following both intraperitoneal administration and addition to BAT homogenates. The *in vitro* effect of 3,5-T2 is plausibly the result of a direct interaction of the iodothyronine with subunit-Va of the COX complex (56), an interaction that was previously reported to prevent the allosteric inhibition exerted by ATP on the enzyme (57). Chronic administration of 3,5-T2 to hypothyroid rats enhanced the expression of UCP1; moreover, in isolated mitochondria, both the inhibition of UCP1-mediated respiration/thermogenesis by GDP and its reactivation by fatty acids, blunted in hypothyroid condition, were enhanced by 3,5-T2 (33). Taken together, these data imply that the 3,5-T2-mediated activation of COX in BAT triggers mitochondrial thermogenesis through the action of UCP1.

3,5-T2 enhances mitochondrial BAT content, and plausibly, PGC-1α is the putative molecular determinant for this effect. Administration of 3,5-T2, in fact, rapidly increases nuclear and mitochondrial PGC-1α levels, indicating a tight coordination between these organelles, hence programming toward mitochondrial biogenesis and thermogenesis (33) (see Figure 2).

3,5-T2 increases the cellular number of nervous fibers that are immune-reactive to tyrosine hydroxylase, a catecholamine-synthesizing enzyme whose expression is related to noradrenergic tone indicating that this iodothyronine increases the sympathetic tone, thus suggesting that part of the thermogenic effect induced by 3,5-T2 in BAT is due to SNS activation. Since adrenergic stimulation of brown adipocytes induces the expression of VEGF [mediated by β-adrenoreceptor/cAMP/PKA signaling pathway (58)], it is plausible that the sympathetic activation promotes the induction of angiogenesis responsible for the higher BAT vascularization observed in 3,5-T2-treated animals (33). The improved BAT vascularization as part of the action of 3,5-T2 on the activation of BAT allows increased blood supply that is crucial to support the higher demand for oxygen and substrates.

## Conclusion

Recent progress in the field concerning the activation of BAT thermogenesis by TH indicates that their metabolic effect both involves their direct action in the target tissue as well as centrally mediated actions through stimulation of specific regions of the brain.

In addition, TH metabolites have emerged as biological active molecules; among these, 3,5-T2 has a calorigenic effect, mimicking the effect induced by T3 on BAT, and emerging data indicate that 3,5-T2 enhances sympathetic tone of BAT.

In relation to this, several questions arise: (i) are endogenous levels of 3,5-T2 relevant to energy balance in normal human physiology, or is 3,5-T2 the basis of a potential pharmacological approach to contrast obesity? (ii) are the effects exerted by T3 on BAT thermogenesis due to T3 itself, or part of these effects are due to its conversion to 3,5-T2?, and (iii) are the thermogenic effects of 3,5-T2 in part mediated centrally?

Unfortunately, studies performed so far do not allow to unambiguously answer the questions above.

Indeed, key experiments concerning the detection of 3,5-T2 serum and tissues levels, in different physiological conditions or following T3 administration are few or lacking. Moreover, despite an extrathyroidal production of 3,5-T2 from T4 have been suggested in humans (59) and in rats an increase in 3,5-T2 serum levels following T3 *in vivo* administration (52) was observed, the identification of the enzyme involved in 3,5-T2 formation from TH has not yet been achieved. It would be a crucial step in the understanding of the basal physiological mechanisms regulating 3,5-T2 availability, and whether some T3 effects are mediated by its conversion into 3,5-T2. New information in this field may reveal future perspectives to allow a deeper understanding of whether 3,5-T2 is a direct stimulator of metabolism with the potential to be an extra level for the regulation of energy metabolism by the thyroid or if it could be used as pharmacological approach to contrast dysmetabolic diseases.

## Author Contributions

FC, AG, ES, and AL contributed with research and writing. FG and AL participated in the conceptual aspect of the mini review and reviewed the article. AL oversaw, assembled, and reviewed the article. FC and AG contributed equally to the study.

## Conflict of Interest Statement

The authors declare that the research was conducted in the absence of any commercial or financial relationships that could be construed as a potential conflict of interest.

## References

[1] Cannon B Nedergaard J. Brown adipose tissue: function and physiological significance. Physiol Rev (2004) 84:277–359. 10.1152/physrev.00015.2003 14715917

[2] Nedergaard J Bengtsson T Cannon B. New powers of brown fat: fighting the metabolic syndrome. Cell Metab (2011) 13(3):238–40. 10.1016/j.cmet.2011.02.009 21356513

[3] Boss O Farmer SR. Recruitment of brown adipose tissue as a therapy for obesity-associated diseases. Front Endocrinol (2012) 3:14. 10.3389/fendo.2012.00014 22654854 PMC3356088

[4] Nedergaard J Bengtsson T Cannon B. Unexpected evidence for active brown adipose tissue in adult humans. Am J Physiol Endocrinol Metab (2007) 293(2):E444–52. 10.1152/ajpendo.00691.2006 17473055

[5] Saito M Okamatsu-Ogura Y Matsushita M Watanabe K Yoneshiro T Nio-Kobayashi J High incidence of metabolically active brown adipose tissue in healthy adult humans: effects of cold exposure and adiposity. Diabetes (2009) 58:1526–31. 10.2337/db09-0530 19401428 PMC2699872

[6] Cannon B Nedergaard J. Yes, even human brown fat is on fire! J Clin Invest (2012) 122(2):486–99. 10.1172/JCI60941 22269320 PMC3266796

[7] van Marken Lichtenbelt WD Vanhommerig JW Smulders NM Drossaerts JM Kemerink GJ Bouvy ND Cold-activated brown adipose tissue in healthy men. N Engl J Med (2009) 360(15):1500–8. 10.1056/NEJMoa0808718 19357405

[8] Bartelt A Bruns OT Reimer R Hohenberg H Ittrich H Peldschus K Brown adipose tissue activity controls triglyceride clearance. Nat Med (2011) 17(2):200–5. 10.1038/nm.2297 21258337

[9] Bartelt A Merkel M Heeren J. A new powerful player in lipoprotein metabolism: brown adipose tissue. J Mol Med (Berl) (2012) 90(8):887–93. 10.1007/s00109-012-0858-3 22231746

[10] Fisher FM Kleiner S Douris N Fox EC Mepani RJ Verdeguer F FGF21 regulates PGC-1α and browning of white adipose tissues in adaptive thermogenesis. Genes Dev (2012) 26(3):271–81. 10.1101/gad.177857.111 22302939 PMC3278894

[11] Shabalina IG Petrovic N de Jong JM Kalinovich AV Cannon B Nedergaard J. UCP1 in brite/beige adipose tissue mitochondria is functionally thermogenic. Cell Rep (2013) 5(5):1196–203. 10.1016/j.celrep.2013.10.044 24290753

[12] Silva JE The thermogenic effect of thyroid hormone and its clinical implications. Ann Intern Med (2003) 139:205–13. 10.7326/0003-4819-139-3-200308050-00010 12899588

[13] Silva JE Thermogenic mechanisms and their hormonal regulation. Physiol Rev (2006) 86(435–464):2006 10.1152/physrev.00009.2005 16601266

[14] Sellers EA You SS Role of the thyroid in metabolic responses to a cold environment. Am J Physiol (1959) 163:81–91. 10.1152/ajplegacy.1950.163.1.81 14771277

[15] Nedergaard J Cannon B. The browning of white adipose tissue: some burning issues. Cell Metab (2014) 20(3):396–407. 10.1016/j.cmet.2014.07.005 25127354

[16] Collins S Yehuda-Shnaidman E Wang H Positive and negative control of Ucp1 gene transcription and the role of beta-adrenergic signaling networks. Int J Obes (Lond) (2010) 34(Suppl 1):S28–33. 10.1038/ijo.2010.180 20935662

[17] Puigserver P. Tissue-specific regulation of metabolic pathways through the transcriptional coactivator PGC1-alpha. Int J Obes (Lond) (2005) 29:S5–9. 10.1038/sj.ijo.0802905 15711583

[18] Leone TC Lehman JJ Finck BN Schaeffer PJ Wende AR Boudina S PGC-1alpha deficiency causes multi-system energy metabolic derangements: muscle dysfunction, abnormal weight control and hepatic steatosis. PLoS Biol (2005) 3(4):e101. 10.1371/journal.pbio.0030101 15760270 PMC1064854

[19] Lin J Wu PH Tarr PT Lindenberg KS St-Pierre J Zhang CY Defects in adaptive energy metabolism with CNS-linked hyperactivity in PGC-1alpha null mice. Cell (2004) 119(1):121–35. 10.1016/j.cell.2004.09.013 15454086

[20] Aquilano K Vigilanza P Baldelli S Pagliei B Rotilio G Ciriolo MR Peroxisome proliferator-activated receptor gamma co-activator 1 alpha (PGC1 alpha) and sirtuin1(SIRT1) reside in mitochondria:possible direct function in mitochondrial biogenesis. J Biol Chem (2010) 28(285):21590–9. 10.1074/jbc.M109.070169 PMC2898414 20448046

[21] Silva JE Catecholamines and the sympathoadrenal system in hypothyroidism. 8th ed In: Braverman LE Utiger RD, editors. Werner and Ingbar’s the Thyroid. Baltimore: Lippincott Williams & Wilkins (2000). p. 820–3.

[22] Silva JE Bianco SD. Thyroid-adrenergic interactions: physiological and clinical implications. Thyroid (2008) 18:157–65. 10.1089/thy.2007.0252 18279016

[23] Pelletier P Gauthier K Sideleva O Samarut J Silva JE. Mice lacking the thyroid hormone receptor-alpha gene spend more energy in thermogenesis, burn more fat, and are less sensitive to high-fat diet-induced obesity. Endocrinology (2008) 149:6471–86. 10.1210/en.2008-0718 18719022

[24] Ribeiro MO Bianco SD Kaneshige M Schultz JJ Cheng SY Bianco AC Expression of uncoupling protein 1 in mouse brown adipose tissue is thyroid hormone receptor-beta isoform specific and required for adaptive thermogenesis. Endocrinology (2010) 51:432–40. 10.1210/en.2009-0667 PMC2817565 19906816

[25] Martinez de Mena R Scanlan TS Obregon MJ. The T3 receptor beta1 isoform regulates UCP1 and D2 deiodinase in rat brown adipocytes. Endocrinology (2010) 151(10):5074–83. 10.1210/en.2010-0533 20719854

[26] Lee JY Takahashi N Yasubuchi M Kim YI Hashizaki H Kim MJ Triiodothyronine induces UCP-1 expression and mitochondrial biogenesis in human adipocytes. Am J Physiol Cell Physiol (2012) 302(2):C463–72. 10.1152/ajpcell.00010.2011 22075692

[27] Bianco AC Silva JE Intracellular conversion of thyroxine to triiodothyronine is required for the optimal thermogenic function of brown adipose tissue. J Clin Invest (1987) 79:295–300. 10.1172/JCI112798 3793928 PMC424048

[28] Bianco AC Silva JE Optimal response of key enzymes and uncoupling protein to cold in BAT depends on local T3 generation. Am J Physiol (1987) 253:E255–63. 10.1152/ajpendo.1987.253.3.E255 3631256

[29] Silva JE. Full expression of uncoupling protein gene requires the concurrence of norepinephrine and triiodothyronine. Mol Endocrinol (1988) 2:706–13. 10.1210/mend-2-8-706 3211156

[30] De Jesus LA Carvalho SD Ribeiro MO Schneider M Kim SW Harney JW The type 2 iodothyronine deiodinase is essential for adaptive thermogenesis in brown adipose tissue. J Clin Invest (2001) 108:1379–85. 10.1172/JCI13803 11696583 PMC209445

[31] Bianco AC Salvatore D Gereben B Berry MJ Larsen PR. Biochemistry, cellular and molecular biology and physiological roles of the iodothyronine selenodeiodinases. Endocr Rev (2002) 23:38–89. 10.1210/edrv.23.1.0455 11844744

[32] Weiner J Kranz M Klöting N Kunath A Steinhoff K Rijntjes E Thyroid hormone status defines brown adipose tissue activity and browning of white adipose tissues in mice. Sci Rep (2016) 12(6):38124. 10.1038/srep38124 27941950 PMC5150531

[33] Lombardi A Senese R De Matteis R Busiello RA Cioffi F Goglia F 3,5-Diiodo-L-thyronine activates brown adipose tissue thermogenesis in hypothyroid rats. PLoS One (2015) 10(2):e0116498. 10.1371/journal.pone.0116498 25658324 PMC4319745

[34] Draman MS Stechman M Scott-Coombes D Dayan CM Rees DA Ludgate M The role of thyrotropin receptor activation in adipogenesis and modulation of fat phenotype. Front Endocrinol (2017) 8:83. 10.3389/fendo.2017.00083 28469599 PMC5395630

[35] Rubio A Raasmaja A Silva JE. Thyroid hormone and norepinephrine signaling in brown adipose tissue. II. Differential effects of thyroid hormone on beta 3-adrenergic receptors in brown and white adipose tissue. Endocrinology (1995) 136:3277–84. 10.1210/endo.136.8.7628361 7628361

[36] Rubio A Raasmaja A Maia AL Kim KR Silva JE Effects of thyroid hormone on norepinephrine signaling in brown adipose tissue. I. Beta 1- and beta 2-adrenergic receptors and cyclic adenosine 3′,5′-monophosphate generation. Endocrinology (1995) 136:3267–76. 10.1210/endo.136.8.7628360 7628360

[37] Ribeiro MO Lebrun FL Christoffolete MA Branco M Crescenzi A Carvalho SD Evidence of UCP1-independent regulation of norepinephrine-induced thermogenesis in brown fat. Am J Physiol Endocrinol Metab (2000) 279:E314–22. 10.1152/ajpendo.2000.279.2.E314 10913031

[38] Carvalho SD Bianco AC Silva JE. Effects of hypothyroidism on brown adipose tissue adenylyl cyclase activity. Endocrinology (1996) 137:5519–29. 10.1210/endo.137.12.8940379 8940379

[39] Lanni A Moreno M Lombardi A Goglia F. Calorigenic effect of diiodothyronines in the rat. J Physiol (1996) 494(Pt 3):831–7. 10.1113/jphysiol.1996.sp021536 8865078 PMC1160681

[40] Weiner J Hankir M Heiker JT Fenske W Krause K Thyroid hormones and browning of adipose tissue. Mol Cell Endocrinol (2017) 458:156–9. 10.1016/j.mce.2017.01.011 28089823

[41] López M Varela L Vázquez MJ Rodríguez-Cuenca S González CR Velagapudi VR Hypothalamic AMPK and fatty acid metabolism mediate thyroid regulation of energy balance. Nat Med (2010) 16(9):1001–8. 10.1038/nm.2207 20802499 PMC2935934

[42] Martínez-Sánchez N Seoane-Collazo P Contreras C Varela L Villarroya J Rial-Pensado E Hypothalamic AMPK-ER stress-JNK1 axis mediates the central actions of thyroid hormones on energy balance. Cell Metab (2017) 26(1):212–29.e12. 10.1016/j.cmet.2017.06.014 28683288 PMC5501726

[43] Martínez-Sánchez N Moreno-Navarrete JM Contreras C Rial-Pensado E Fernø J Nogueiras R Thyroid hormones induce browning of white fat. J Endocrinol (2017) 232(2):351–62. 10.1530/JOE-16-0425 27913573 PMC5292977

[44] Ruderman NB Saha AK. Metabolic syndrome: adenosine monophosphate-activated protein kinase and malonyl coenzyme A. Obesity (2006) 14(Suppl 1):25S–33S. 10.1038/oby.2006.279 16642960

[45] Contreras C González-García I Martínez-Sánchez N Seoane-Collazo P Jacas J Morgan DA Central ceramide-induced hypothalamic lipotoxicity and ER stress regulate energy balance. Cell Rep (2014) 9(1):366–77. 10.1016/j.celrep.2014.08.057 25284795 PMC5157160

[46] Alvarez-Crespo M Csikasz RI Martínez-Sánchez N Diéguez C Cannon B Nedergaard J Essential role of UCP1 modulating the central effects of thyroid hormones on energy balance. Mol Metab (2016) 5(4):271–82. 10.1016/j.molmet.2016.01.008 27069867 PMC4812006

[47] Goglia F Biological effects of 3,5-diiodothyronine-T(2). Biochemistry (Mosc) (2005) 70:164–72. 10.1007/s10541-005-0097-0 15807655

[48] Goglia F The effects of 3,5-diiodothyronine on energy balance. Front Physiol (2015) 13(5):528 10.3389/fphys.2014.00528 PMC4292545 25628573

[49] Antonelli A Fallahi P Ferrari SM Di Domenicantonio A Moreno M Lanni A 3,5-Diiodo-L-thyronine increases resting metabolic rate and reduces body weight without undesirable side effects. J Biol Regul Homeost Agents (2011) 25(4):655–60. 22217997

[50] Davis PJ Goglia F Leonard JL Non-genomic actions of thyroid hormone. Nat Rev Endocrinol (2015) 12(2):111–21. 10.1038/nrendo.2015.205 26668118

[51] Moreno M Lanni A Lombardi A Goglia F How the thyroid controls metabolism in the rat: different roles for riiodothyronine and diiodothyronines. J Physiol (1997) 505:529–38. 10.1111/j.1469-7793.1997.529bb.x 9423191 PMC1160082

[52] Moreno M Lombardi A Beneduce L Silvestri E Pinna G Goglia F Are the effects of T3 on resting metabolic rate in euthyroid rats entirely caused by T3 itself? Endocrinology (2002) 143(2):504–10. 10.1210/endo.143.2.8613 11796504

[53] Lombardi A De Matteis R Moreno M Napolitano L Busiello RA Senese R Responses of skeletal muscle lipid metabolism in rat gastrocnemius to hypothyroidism and iodothyronine administration: a putative role for FAT/CD36. Am J Physiol Endocrinol Metab (2012) 303(10):E1222–33. 10.1152/ajpendo.00037.2012 22967501

[54] Padron AS Neto RA Pantaleão TU de Souza dos Santos MC Araujo RL de Andrade BM Administration of 3,5-diiodothyronine (3,5-T2) causes central hypothyroidism and stimulates thyroid-sensitive tissues. J Endocrinol (2014) 21(3):415–27. 10.1530/JOE-13-0502 24692290 PMC4045230

[55] Lanni A Moreno M Lombardi A Goglia F 3,5-Diiodo-L-thyronine and 3,5,30-triiodo-L-thyronine both improve the cold tolerance of hypothyroid rats, but possibly via different mechanisms. Pflugers Arch (1998) 436(3):407–14. 10.1007/s004240050650 9644223

[56] Goglia F Lanni A Barth J Kadenbach B. Interaction of diiodothyronines with isolated cytochrome c oxidase. FEBS Lett (1994) 346(2–3):295–8. 10.1016/0014-5793(94)00476-5 8013649

[57] Arnold S Goglia F Kadenbach B. 3,5-Diiodothyronine binds to subunit Va of cytochrome-c oxidase and abolishes the allosteric inhibition of respiration by ATP. Eur J Biochem (1998) 252(2):325–30. 10.1046/j.1432-1327.1998.2520325.x 9523704

[58] Fredriksson JM Lindquist JM Bronnikov GE Nedergaard J. Norepinephrine induces vascular endothelial growth factor gene expression in brown adipocytesthrough a beta-adrenoreceptor/cAMP/protein kinase A pathway involving Src but independently of Erk1/2. J Biol Chem (2000) 275(18):13802–11. 10.1074/jbc.275.18.13802 10788502

[59] Lehmphul I Brabant G Wallaschofski H Ruchala M Strasburger CJ Köhrle J Detection of 3,5-diiodothyronine in sera of patients with altered thyroid status using a new monoclonal antibody-based chemiluminescence immunoassay. Thyroid (2014) 24:1350–60. 10.1089/thy.2013.0688 24967815

